# Mechanical Influences on Morphogenesis of the Knee Joint Revealed through Morphological, Molecular and Computational Analysis of Immobilised Embryos

**DOI:** 10.1371/journal.pone.0017526

**Published:** 2011-02-28

**Authors:** Karen A. Roddy, Patrick J. Prendergast, Paula Murphy

**Affiliations:** 1 Department of Zoology, School of Natural Sciences, Trinity College Dublin, Dublin, Ireland; 2 Trinity Centre for Bioengineering, School of Engineering, Trinity College Dublin, Dublin, Ireland; Ohio State University, United States of America

## Abstract

Very little is known about the regulation of morphogenesis in synovial joints. Mechanical forces generated from muscle contractions are required for normal development of several aspects of normal skeletogenesis. Here we show that biophysical stimuli generated by muscle contractions impact multiple events during chick knee joint morphogenesis influencing differential growth of the skeletal rudiment epiphyses and patterning of the emerging tissues in the joint interzone. Immobilisation of chick embryos was achieved through treatment with the neuromuscular blocking agent Decamethonium Bromide. The effects on development of the knee joint were examined using a combination of computational modelling to predict alterations in biophysical stimuli, detailed morphometric analysis of 3D digital representations, cell proliferation assays and in situ hybridisation to examine the expression of a selected panel of genes known to regulate joint development. This work revealed the precise changes to shape, particularly in the distal femur, that occur in an altered mechanical environment, corresponding to predicted changes in the spatial and dynamic patterns of mechanical stimuli and region specific changes in cell proliferation rates. In addition, we show altered patterning of the emerging tissues of the joint interzone with the loss of clearly defined and organised cell territories revealed by loss of characteristic interzone gene expression and abnormal expression of cartilage markers. This work shows that local dynamic patterns of biophysical stimuli generated from muscle contractions in the embryo act as a source of positional information guiding patterning and morphogenesis of the developing knee joint.

## Introduction

Each skeletal rudiment and joint of the limb can be identified by its unique, species specific, size and shape. These individual shapes emerge by the local modulation of cellular processes, such as cell proliferation, differentiation, extracellular matrix synthesis, cell shape and size [1], creating complex shapes from relatively simple initial morphologies. Skeletal morphogenesis is regulated by a combination of inductive regulatory signals produced by the constituent tissues [reviewed in 2,3,4]. While such networks of molecular regulatory signals are clearly essential to the correct establishment of spatial patterning, there is evidence that features of the physical environment, such as mechanical forces induced by muscle contraction, contribute to regulatory mechanisms governing morphogenesis. We focus on the developing chick knee joint as a convenient model to investigate how mechanical forces integrate with cellular and molecular events to impact the emerging properties of the skeleton.

We have previously shown that the complex 3D shape of the knee joint emerges following the initiation of muscle contractions, between chick embryonic stages Hamburger and Hamilton (HH)28 and HH34 [5]. The interfacing ends of the cartilaginous rudiments (including the prominent condyles of the distal femur) dictate the shape of the articular surfaces of the knee while other joint structures such as articular cartilages, menisci and the synovium derive from cells in the joint interzone [6], [7]. The emergence of knee joint shape and form must therefore involve the local regulation of growth in the cartilaginous rudiments and tissue differentiation within the joint interzone.

Several lines of evidence show that contraction of the developing embryonic musculature is required for normal skeletogenesis. Human congenital malformations [8], [9] and animal models where muscle contractions are removed or altered using neuromuscular blocking agents [10], [11], [12], [13], surgery [11] or explant culture [14], [15], [16] and mouse mutants where no skeletal muscle forms [17], [18], [19], [20], lead to underdeveloped, brittle and in some cases misshapen skeletal elements [11], [12], [21], [22]. Joints appear to be particularly sensitive with immobilisation leading to loss of joint structures such as the cavity, articular surfaces and patella [10], [11], [12], [19], [20], [21], [23]. It is unknown how mechanical stimulation derived from movement can influence rudiment and joint morphogenesis but computational modelling has been used to predict mechanical loads acting on the tissues. Previous studies used Finite Element (FE) modelling to predict mechanical forces in simplified representations of the skeleton to investigate joint formation [24], endochondral ossification [25], [26], the emergence of the femoral bicondylar angle [27] and developmental dysplasia of the hip [28]. We previously [29] created a FE model from morphologically accurate 3D data captured from the developing chick tibiotarsus to simulate the dynamic patterns of stimuli generated by muscle contraction. A striking correspondence between the patterns of stimuli and the dynamics of ossification was noted and we further showed that in an altered mechanical environment ossification was reduced and the *in vivo* expression of a number of genes involved in bone formation was altered [13]. More recently we used a similar approach to create a morphologically accurate FE model of knee joint development [30], indicating that the tempero-spatial pattern of mechanical stimuli generated in the distal femur by muscle contraction corresponds with aspects of the pattern of shape changes and with differential rates of cell proliferation in the femoral condyles. This led to the proposal that mechanical forces could act as a physical form of positional information, generating local patterns that modulate cellular events such as cell proliferation and differentiation, thereby guiding tissue morphogenesis.

A number of key molecules regulating cartilage growth and differentiation [31], [32], [33], [34], [35], [36] and joint formation [32], [37], [38], [39], [40], [41] have been identified. PTHLP(also known as PTHrP) has been shown to act in a regulatory loop with Ihh to maintain a pool of proliferating chondrocytes at the epiphysis of long bones [34], [42]. BMP and FGF family members act in an antagonistic relationship to co-ordinate differentiation and proliferation processes during development of the skeletal rudiment [43]. A large number of genes, including BMP2, FGF2, FGFR2, PTHLH, β1 integrin (ITGB1), CD44 and HAS2, are expressed specifically in the joint region. Several of these gene products regulate chondroctye growth and differentiation while others such as CD44 and HAS2 regulate joint cavitation through the action of hyaluronan [44], [45], [46]. CD44 encodes one of the major receptors for hyaluronan while HAS2 encodes an enzyme involved in its synthesis. Inhibition of α5β1 integrin leads to ectopic joint formation while missexpression causes the inhibition of joint formation leading to fused long bones [47].

Very little is known about how cells respond to mechanoregulation, especially in an *in vivo* developing system. Mechanoregulation of chondrocyte proliferation and biosynthesis has been extensively studied in a wide range of culture systems, including explants, monolayer and 3D scaffolds [reviewed in 48] where it has been proposed that continuous loads or high frequency, high magnitude loads inhibit cell matrix synthesis and growth [49], [50], [51], [52] while low magnitude dynamic loading stimulates matrix synthesis [49], [50], [51], [53]. For example, the dynamic compression of cartilage explants by approximately 3% was shown to stimulate matrix synthesis while graded levels of static compression did not [49]. Mechanical forces are known to influence the expression of certain genes in mechanically stimulated cells when compared to non stimulated cells [52], [54], [55]. Such genes have been called mechanosensitive or mechanoresponsive and evidence exists that several of the molecules involved in the development of the joint and regulation of ossification are mechanosensitive, at least in a cell culture context. Such genes include the previously mentioned BMP2, CD44, β1 integrin subunit, FGF2, FGFR2 and PTHLP (Table 1). A limited number of studies have investigated alterations in the expression patterns of regulatory genes in developing tissues *in vivo* in response to immobilisation, showing for example alteration in FGF2 [37], IHH and COLX [22]. Such *in vivo* studies have the advantage of demonstrating mechanosensitivity within a specific developmental context and also make it possible to relate the changes in gene expression to changes in tissue differentiation and morphogenesis.

**Table 1:** Summary of regulatory genes selected for analysis based on functional evidence and mechanosensitivity.

Gene	Evidence of skeletal function	Evidence of mechanosensitivity
BMP2	chondrocyte maturation and proliferation [31], [35], [85],	Distraction osteogenesis (*in vivo*) [86]
CD44	joint cavity formation [44]	in culture [71], [87]
HAS 2	joint cavity formation [88]	no evidence
β1 integrin	interzone formation [47]	in explants [89]
WNT9a	interzone specification [90], [91]	no evidence
PTHLP	maintains chondrocyte proliferation [31], [34], [42]	in culture [92]
FGF2	joint cavity formation [93], chondroctye maturation [43], [94]	*in vivo* [93]
FGFR2	proliferation of osteoprogenitor cells [95]	in culture [96]
COL2A1	ECM matrix component [97], marker of proliferating chondrocyes	*in vivo* [20]
TNC	Articular cartilage ECM matrix component [98], [99]	*in vivo* [98]

In this paper we further explore the link between local patterns of biophysical stimuli generated by embryonic muscle contractions and the generation of shape and structure in the avian knee joint. *In ovo* immobilisation was used to alter the mechanical environment during development and changes in the resulting structure and shape of the knee joint region were revealed. Shape in particular was analysed following 3D imaging of control and immobilised specimens using Optical Projection Tomography (OPT). To explore cellular processes impacted by mechanical stimulation, patterns of cell proliferation in the distal femur were compared between immobilised and control specimens. A FE model of rigid muscle paralysis was used to determine how the local mechanical information produced by muscle contractions would be altered by paralysis and in turn how this compares with the alterations in joint shape and cell proliferation observed. Finally the effect of altered mechanical forces on the expression of regulatory genes was investigated using in situ hybridisation. Genes were chosen for analysis based on previous experimental evidence of a regulatory role in the process of joint formation and an indication of mechanosensitivity in another cellular context (Table 1). The findings support and further the hypothesis that patterns of biophysical stimuli generated by the contracting musculature act as a type of positional information during skeletal morphogenesis, impacting the molecular regulation of cell proliferation and tissue patterning.

## Results

### Abnormal development of the knee joint following muscle immobilisation

**Figure 1:**
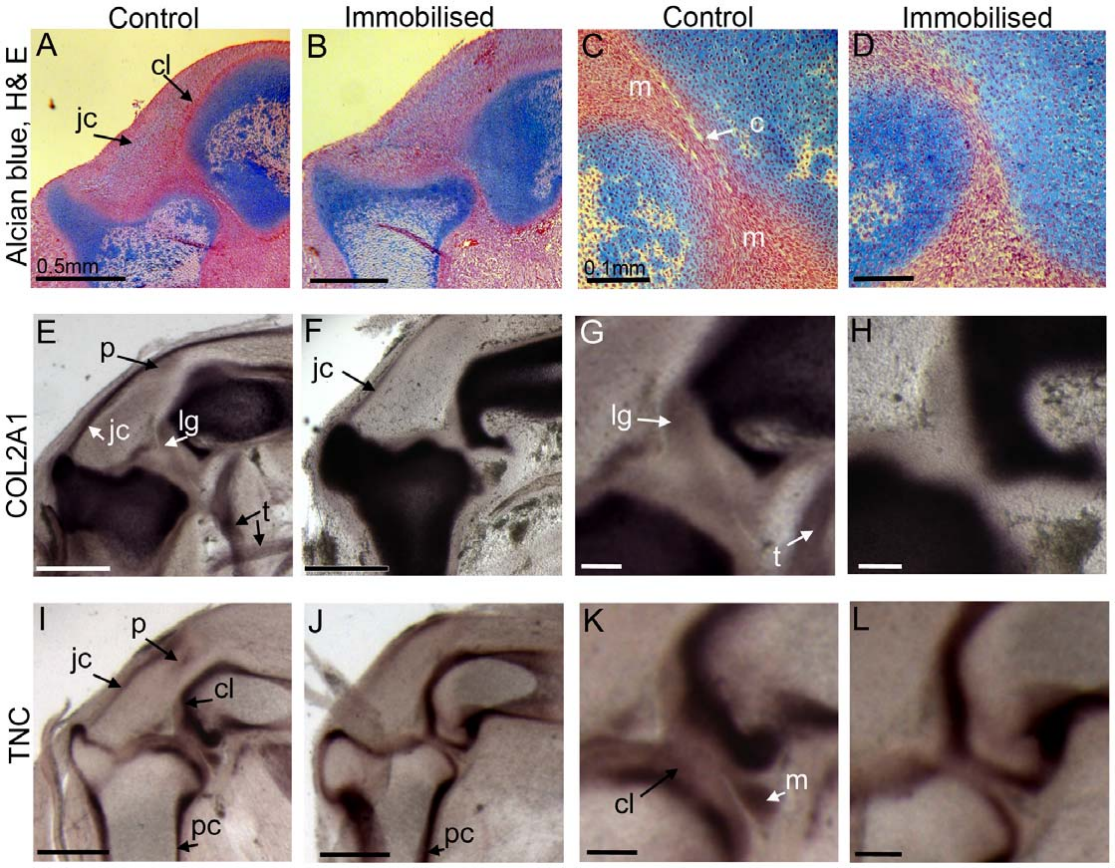
Anatomical changes in the knee joints of immobilised embryos. Longitudinal sections through the chick knee joint of control and immobilised embryos (4.5 days of immobilisation) at low (A, B, E, F, I, J) and high magnification (C, D, G, H, K, L). Histological sections (E–D) were stained using alcian blue and counter stained with haematoxlyin and eosin. Other sections show the expression of COL2A1 and TNC mRNA in control and immobilised knee joints. c; cavity, cl; chondrogenous layers, jc; joint capsule, lg ; ligament, m; meniscus, p; patella, pc ; perichondrium, t; tendon. Scale bar 0.5 mm (A, B, E, F, I, J) and 0.1 mm (C, D, G, H, K, L).

Comparison of embryos immobilised with 0.5% DMB for 4–5 days, commencing on day 4.5 of incubation, and control specimens, revealed a number of consistent abnormalities. Staging of the embryos, using the Hamburger and Hamilton criteria [56], insured that only stage matched specimens were compared. Drug treated embryos showed previously reported effects of immobilisation including spinal curvature and joint contracture (not shown) [10], [21]. Specifically in the knee joint, histological sections showed a general reduction in the separation of the rudiments, altered cellular organisation in the interzone with no clear definition of chondrogenous layers and no sign of cavitation in the altered mechanical environment of immobilised specimens (Figure 1A–D and K,L). Additional alterations to knee joint associated tissues were revealed through marker gene expression analysis. Collagen type II alpha1 (COL2A1) gene expression marks the joint capsule, developing ligaments and tendons and initial appearance of the patella, in addition to the cartilaginous rudiments in control specimens at this stage (Figure 1E and G). Tenascin C (TNC) expression also marks the joint capsule and patella and reveals the chondrogenous layers, the perichondrium and the appearance of the menisci in the joint interzone (Figure 1I and K). In immobilised knee joints, expression analysis of these tissue markers revealed absence of the inter-articular ligaments (Figure. 1F and H), the chondrogenous layers and menisci (Figure 1J and L). The expression of both markers also appeared to be reduced or absent in the joint capsule and patella region (Figure 1F, J) in immobilised joints.

### Shape changes in the knee joint

**Figure 2:**
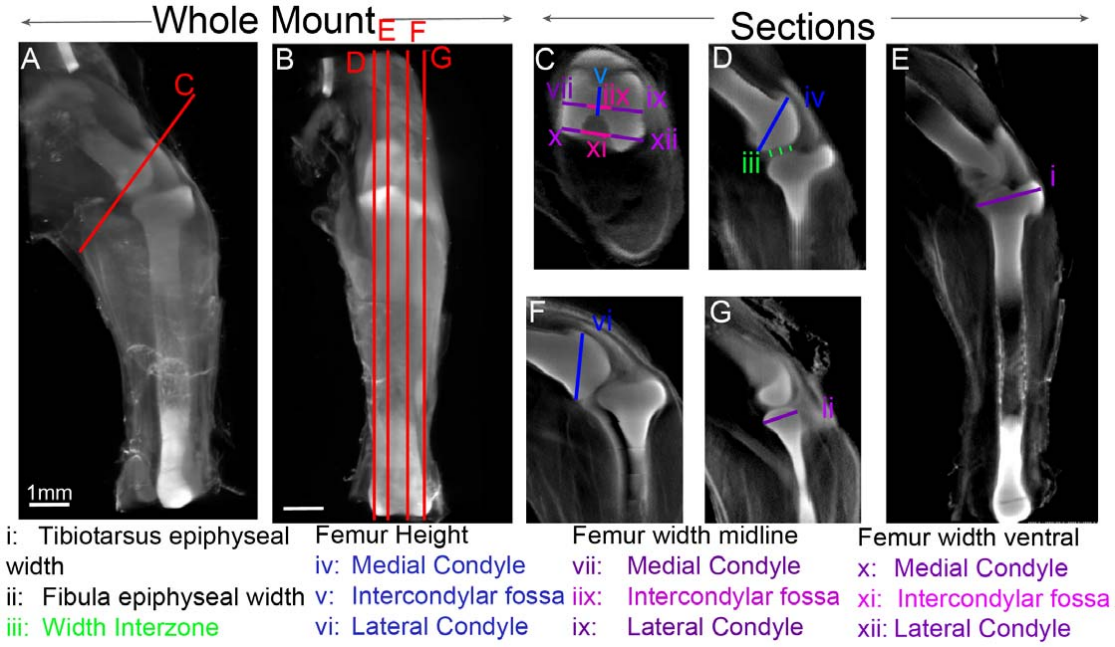
Overview of morphometric analysis of the knee joint. 3D volume representation of the hind limb (A–B) at HH35 with guidelines in red shows the location of consistent virtual sections taken through the hindlimbs of all specimens (C–G). Individual measurements are indicated by lines i-xii. Scale bar 1 mm.

To reveal shape changes in the knee joint following immobilisation, 3D analysis of Alcian blue stained, OPT scanned specimens following 4 or 5 days of immobilisation was carried out (n = 17 and 32 for immobilised specimens on days 4 and 5, n = 16 and 18 for controls). 3D digital representations of each knee joint specimen could be oriented to view comparable sections [57] and take measurements that capture characteristic aspects of shape including the width of the proximal tibiotarsus and fibula, the separation of the tibiotarsus and femur (interzone) and the height and width of the condyles and intercondylar fossa of the distal femur (individual measurements detailed in Figure 2). Statistical analysis of the measurements showed that immobilisation had a significant effect on specific morphological features of the knee (Table 2). Immobilisation caused a significant reduction in the width of the proximal epiphysis of the tibiotarsus and fibula (Table 2). The distal end of the femur, showed a reduction in the height of both condyles in the dorso-ventral orientation but no significant reduction in width following 4 days of immobilisation (Table 2), either at the midline or ventral aspect. The same overall effects on shape were seen following 4 and 5 days of immobilisation except that a reduction in the width of condyles in the ventral aspect became apparent with extended treatment (Table 2, highlighted).

**Table 2:** Comparison of mean morphometric measurements of control and immobilised knee joints following immobilisation for 4 or 5 days.

		Day 4				Day 5			
		Control	Immob	%reduction	significance	Control	Immob	%reduction	significance
**Tibiotarsus** Epiphyseal width (i)		1.18	0.94	19.65	F_(1,29)_ = 60.43, *<0.001*	1.19	1.01	15.06	F_(1,46)_ = 62.75, *p<0.001*
**Fibula** Epiphyseal width (ii)		0.55	0.45	18.44	F_(1,29)_ = 25.61,*p<0.001*	0.57	0.46	20.29	F_(1,46)_ = 90.92, *p<0.001*
**Interzone** (iii)		0.08	0.06	30.12	F_(1,29)_ = 35.55, p*<0.001*	0.10	0.07	35.59	F_(1,46)_ = 41.61, *p<0.001*
**Femur** Height	Lateral Condyle (iv)	0.96	0.79	18.26	F_(1,29)_ = 36.40, *p<0.001*	1.02	0.88	13.22	F^(1,46)^ = 58.62, *p<0.001*
	Intercondylar fossa (iv)	0.30	0.27	10.33	F_(1,29)_ = 4.19, *p = 0.05*	0.32	0.30	6.64	F_(1,46)_ = 7.93, *p = 0.007*
	Medial Condyle (v)	0.81	0.70	12.98	F_(1,29)_ = 23.22, *p<0.001*	0.82	0.72	12.49	F_(1,46)_ = 23.37, *p<0.001*
**Femur** Width midline	Lateral Condyle (vi)	0.40	0.39	NS		0.46	0.43	NS	
	Intercondylar fossa (vii)	**0.29**	**0.17**	**41.63**	**F_(1,29)_ = 36.67,** ***p<0.001***	**0.25**	**0.18**	**30.18**	**F_(1,46)_ = 28.51, ** ***p<0.001***
	Medial Condyle (iix)	0.30	0.30	NS		0.34	0.32	NS	
**Femur** Width Ventral	Lateral Condyle (ix)	0.42	0.39	NS		**0.50**	**0.42**	**15.41**	**F_(1,46)_ = 27.46, ** ***p<0.001***
	Intercondylar fossa (x)	**0.31**	**0.17**	**44.69**	**F_(1,29)_ = 18.40,** ***p<0.001***	**0.31**	**0.20**	**35.95**	**F_(1,46)_ = 98.37, ** ***p<0.001***
	Medial Condyle (xi)	0.29	0.27	NS		**0.33**	**0.28**	**13.88**	**F_(1,46)_ = 24.18, ** ***p<0.001***

A particularly interesting pattern with respect to the width of the intercondylar fossa is highlighted in bold. Percentage differences between the mean lengths of the measurements are shown with the associated statistical significance.

The apparent reduction in the separation of rudiments in the knee joint noted from histological sections was confirmed here through a significant reduction in the size of the interzone separating the femur and the tibiotarsus; 30.1% and 35.6% following 4 and 5 days of treatments respectively (p<0.001, Table 2).

The strongest and most consistent change in shape was seen in the width of the intercondylar fossa; reduced by 41.6% (P<0.001) at the midline and 44.7% (p<0.001) at the ventral side of the femur following 4 days of immobilisation (Table 2). This reduction in the separation of the femoral condyles is still obvious after 5 days of immobilisation (30.2 and 36%, p<0.001).

**Figure 3:**
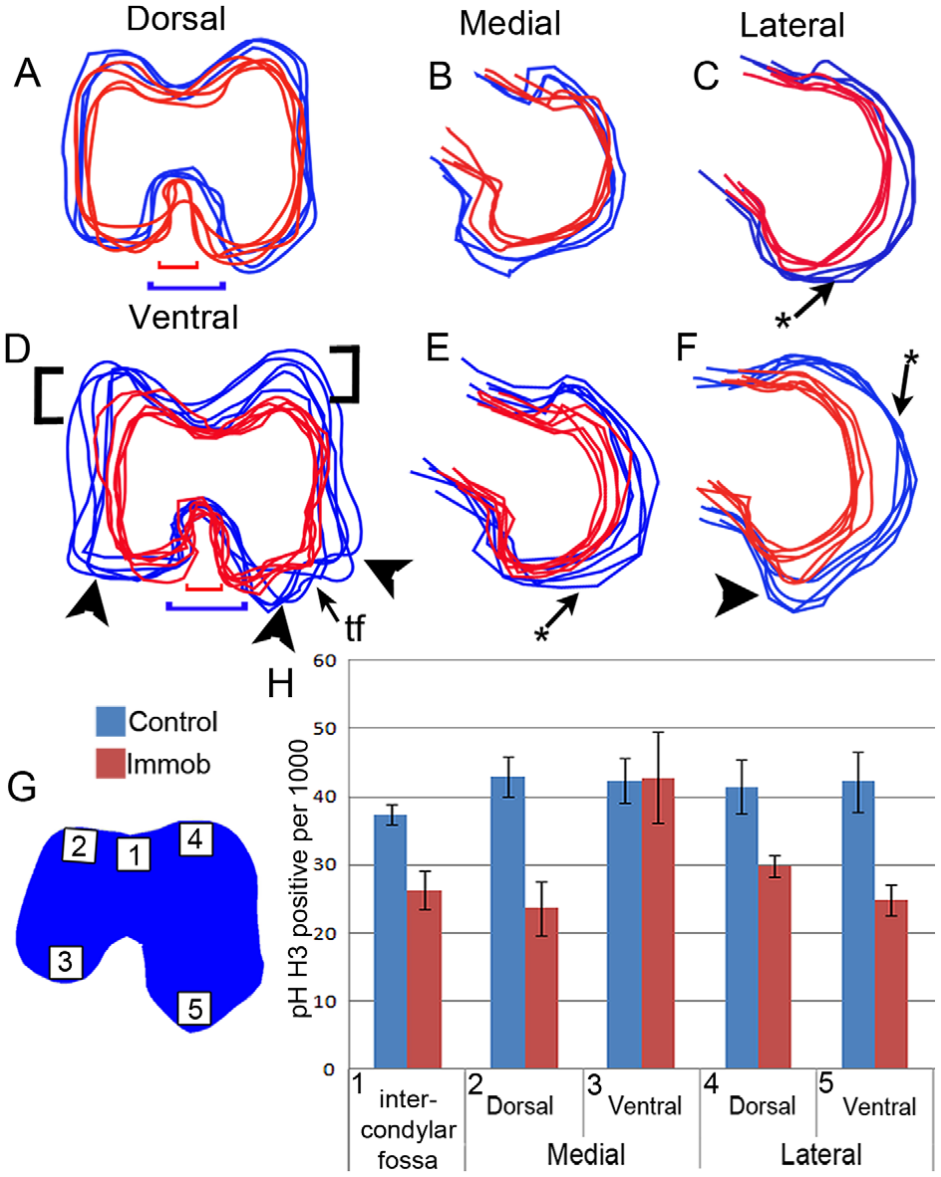
Comparison of cartilage shape and cell proliferation in distal femora of control (blue) and immobilised (red) embryos. Outlines of the cartilage anlaga (A–F) were extracted from virtual sections (Fig.2, C, D, F). A minimum of four outlines per treatment were overlaid. Arrow heads indicate region of reduced outgrowth in the immobilised animals. *indicates flattening of rudiment surfaces. Black brackets show extent of growth reductions in the dorsal aspect of the condyles. Coloured brackets compare width of the intercondylar fossa of control (red) and immobilised (blue) embryos (A, D). The distribution of proliferating chondroctyes in the femur across 5 regions, represented in G (boxes 1–5), was compared in control (blue) and immobilised (red) embryos (h). tf; trochlea fibularis, IF; intercondylar fossa.

This and other detailed aspects of local shape changes in the distal femur were visualised by outlining cartilage (alcian blue stained) in comparable sections of 3D reconstructions. Outlines were generated using the sections shown in Figure 2 C, D and F and physically overlaid so that the medial and lateral sides of the femora in the sections were parallel and the midpoints of the intercondylar fossa were overlapping. Overlaying outlines of this characteristic view of control and immobilised specimens highlighted the effect of rigid paralysis on the emergence of shape in the femoral condyles (Figure 3A–F). Without muscle contraction the general shape of the knee joint is much simpler, joint surfaces are flattened (e.g. flattening of the lateral condyle shown in Figure 3C and F) and functional outgrowths are lost. After 4 days of immobilisation the reduction in width of the intercondylar fossa was very obvious, particularly ventrally (Figure 3A). The surface of the lateral condyle also appeared to be flattened by immobilisation (Figure 3C). Five days of immobilisation caused greater simplification. In particular note the reduction in height of the medial and lateral condyles in the dorsal aspect (Figure 3D, brackets) and the reduced outgrowths on the ventral aspect of the condyles (arrowheads Figure 3D) in the region of the trochlea fibularis grove where the femur interfaces with the fibula enabling smooth movement in later life. A flattening of the articular surfaces was now apparent in both condyles (Figure 3E,F).

### Alteration of cell proliferation patterns in immobilised specimens

A comparison of the proportion of proliferating cells in five selected locations of the distal femur in control and experimentally immobilised embryos (4 days of immobilisation) was performed (Figure 3G,H). Looking across the locations in control animals, similar proportions of proliferating cells were observed across the femur head except for a slightly lower proportion in the region adjacent to the intercondylar fossa. The effect of treatment on cell proliferation was investigated using a generalised linear mixed effects model where multiple sections were nested within individuals in order to take account of the nested nature of the data. Combining data across the locations, treatment significantly reduced the proportion of proliferating cells by an average of 11.8/1000 chondrocytes (s.e. = 3.7, df = 4, p = 0.03) (not shown). Examining each location separately, immobilisation caused a significant reduction in the proportion of proliferating cells in four out of the five locations with the ventral aspect of the medial condyle being the only region unaffected (Figure 3H). These changes correspond to the major alterations observed in joint shape following immobilisation with large reductions in the dorsal side of both condyles and no apparent reduction in the ventral aspect of the medial condyle (Figure 3D). Narrowing of the intercondylar fossa was one of the strongest effects of immobilisation revealed by the morphometric study (Table 2) and the proportion of proliferating cells was reduced in the cartilage rudiment adjacent to the dorsal aspect of the fossa.

### Effect of immobilisation on patterns of biophysical stimuli in the developing knee

**Figure 4:**
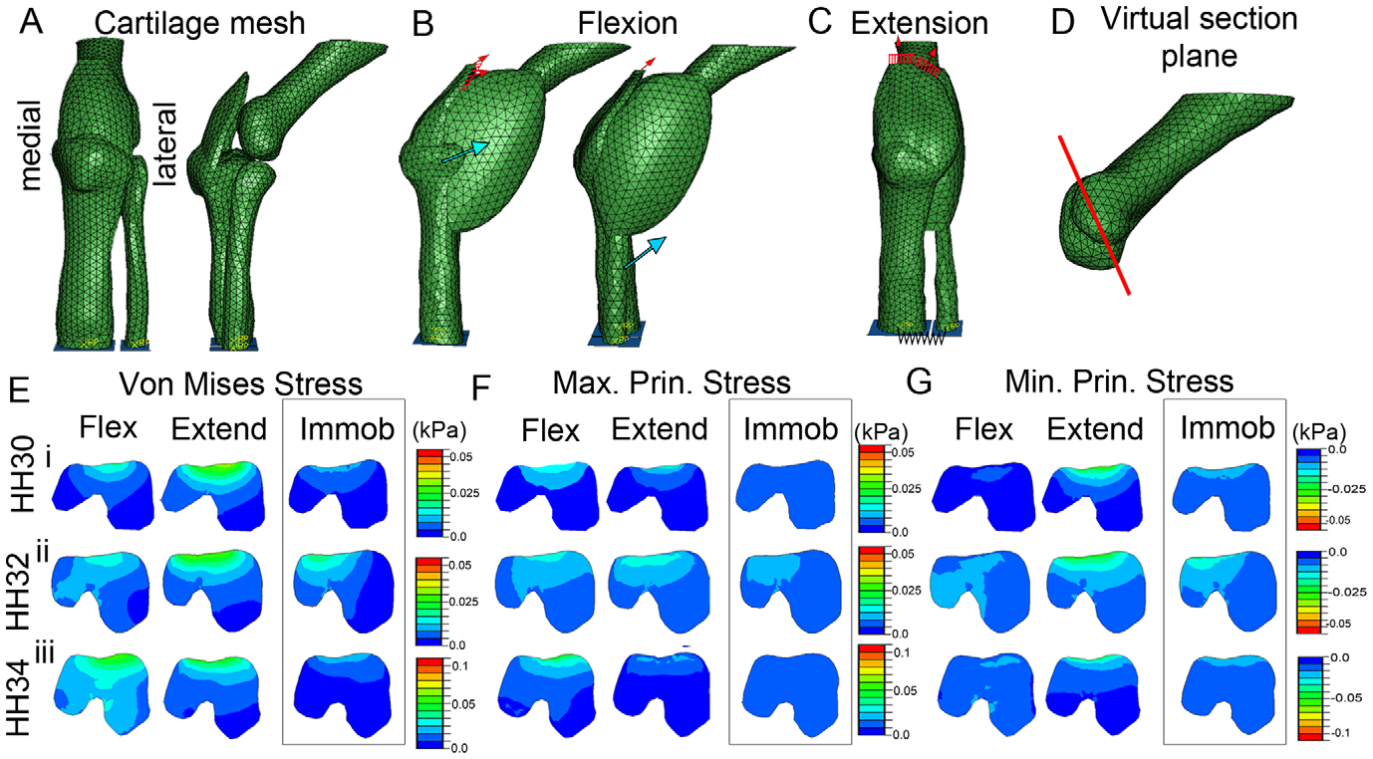
Predicted patterns of biophysical stimuli in FE models of immobilised (boxed, right column of each panel) compared to normal developing femora. Illustrations show the FE mesh (A), the placement of the loads for both flexion (B) and extension (C) and the plane of section (D) shown in E, F and G. The patterns of Von Mises stress (E), Maximum (tension) (F) and Minimum (compression) (G) Principal stress, were captured in equivalent sections through the distal femur (indicated in D) at HH30 (row i), HH32 (ii) and HH34 (iii). Patterns at mid flexion and extension are shown for the normal models whereas the constant pattern during rigid paralysis is shown for immobilised. Stimuli patterns for normal contractions are captured from simulations previously described [30].

**Figure 5:**
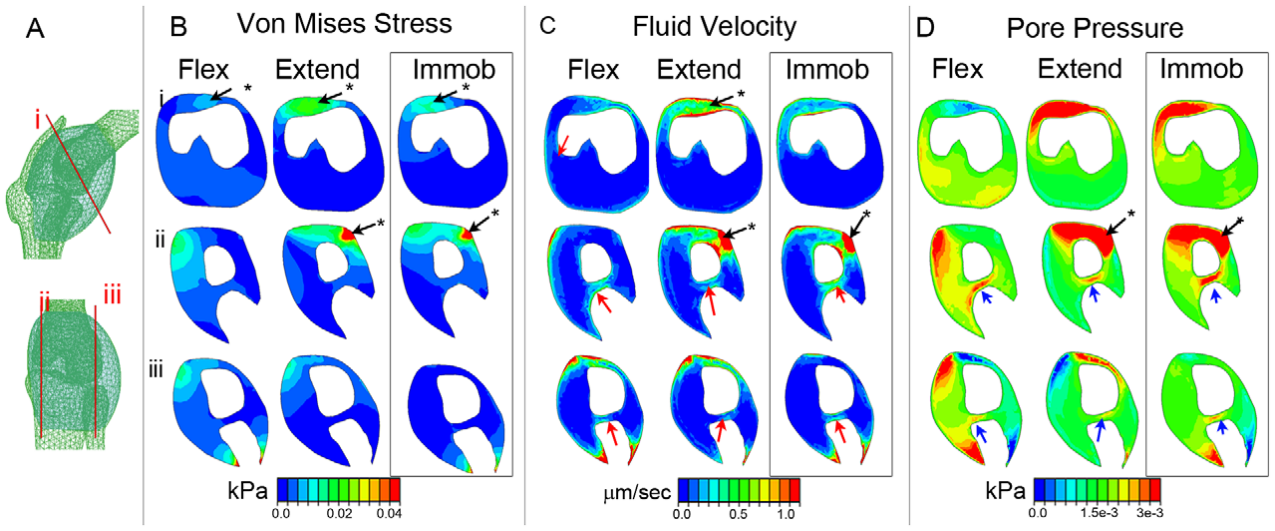
Comparison of the Von Mises Stress (B), Fluid Velocity (C) and Pore Pressure (D) within sections of the developing knee interzone region at HH32 under rigid paralysis (boxed on right) and normal muscle contractions. Location of sections, is indicated by red lines in A. * indicates location of the patella region (B), red arrow indicates pattern of elevated fluid velocity in the presumptive chondrogenous layer (C), blue arrows indicate peak in pore pressure at the intermediate layer (D). Flex; mid flexion, Extend; mid extension, Immob; Immobilisation. The images for normal contractions are captured from simulations previously described [30].

We previously constructed a FE model to predict biophysical stimuli during an extension/flexion cycle of muscle contraction in the developing knee joint region [30]. We adapted this model to represent rigid limb paralysis (simultaneous contraction of all muscles), as induced by DMB, to compare predicted biophysical stimuli in immobilised as compared to normal developing joints. The obvious change to the mechanical environment in the rigid model is the loss of dynamic stimulation associated with a contraction cycle since muscle tension is constant. In addition, changes in the predicted patterns of the local mechanical environment are indicated, including reductions to the magnitude of loads and a general simplification of the spatial pattern of stimuli (Figures 4 and 5).

Focusing on the distal femur (Figure 4), the most obvious pattern of stimuli observed in the normal models was the distinct peak centred on the region adjacent to the intercondylar fossa and a general elevation of stimuli in the dorsal regions of both condyles (Figure 4E–G, Flex, Extend). While this peak was still predicted for patterns of Von Mises stress and Minimum Principal stress (compression) (Figure 4E, G) under rigid paralysis, it is reduced for patterns of Maximum Principal stress (tension) (Figure 4F). Thus, while the normal joint develops under the influence of dynamic patterns of tension and compression (compare Flex and Extend for each) the rigid paralysis model is largely experiencing compression.

### Biophysical stimuli patterns in the interzone region correspond with the location of anatomical features that are disrupted following rigid paralysis

The FE simulations of biophysical stimuli in the developing chick knee joint comparing normal muscle contraction and rigid paralysis, as described above, were also used to examine the mechanical environment in the interzone region between the skeletal rudiments (Figure 5). The models were generated using the first direct estimates of mechanical properties of cartilage and interzone tissue in the developing chick knee using nanoindentation, as previously described [30]. The general pattern of stimuli did not vary between stages, thus Figure 5 presents the results at HH32 only, the midpoint in the study.

The patterns of stimuli predicted under normal muscle contractions indicated that several territories and tissues developing within the interzone experience specific patterns of stimulation [initially described in 30, Figure 5]. While the patella normally develops under dynamic magnitudes of stress, fluid velocity and pore pressure (Figure 5B–D, indicated by *; compare Flex and Extend), in rigid paralysis, the territory of elevated stimulation is restricted and the dynamic aspect is lost. This is of particular note since the patella fails to appear in immobilised embryos (Figure 1). Appearance of the chondrogenous layers is also lost in immobilised embryos. The chondrogenous layers emerge in a location that experiences dynamic patterns of elevated fluid velocity (Figure 5C. indicated by red arrow) while the intermediate layer, separating the two chondrogenous layers, emerges under a pattern of dynamically elevated pore pressure (Figure 5D, indicated by blue arrow). Under rigid paralysis, elevated fluid velocity in the presumptive chondrogenous layer and elevated pore pressure in the presumptive intermediate layer is still predicted (Figure 5, C,D), although in less extensive territories, but the pattern is no longer dynamic.

### The expression of genes that regulate joint morphogenesis is altered in immobilised embryos

**Figure 6:**
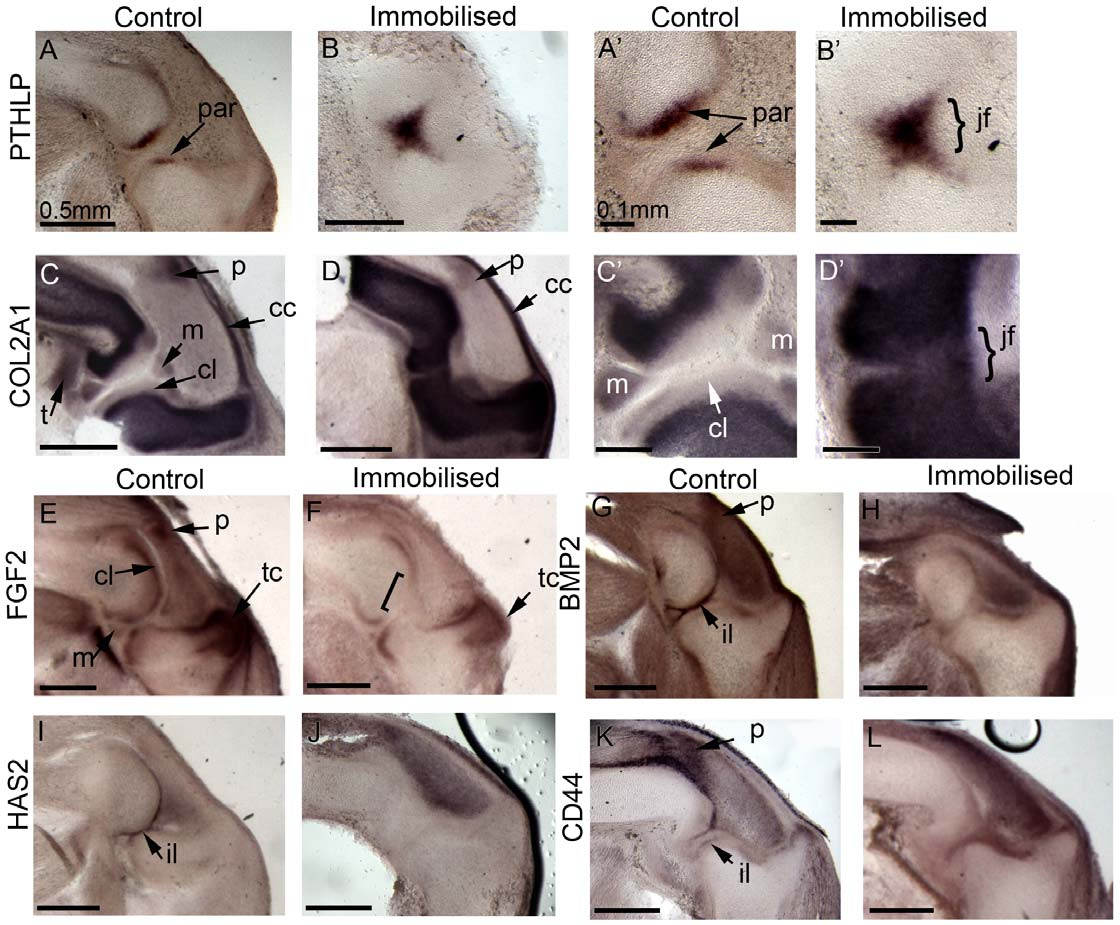
Expression of candidate mechanosensitive genes in control and immobilised specimens on longitudinal sections through the knee joint. Images A’–D’ (scale bar 0.1 mm) show the knee region of A to D (scale bar 0.5 mm) at a higher magnification. cc; capsular condensation, cl; chondrogenous layer, iz; intermediate layer, jf; joint fusion, m; meniscus, p; patella, par; periarticular cartilage, t; tendon, tc; tibial crest.

Specific changes were observed in the shape of cartilage rudiments and the appearance of tissues associated with the knee joint in immobilised embryos (Figures 1 and 3). To explore the molecular basis of these changes, a number of regulatory genes implicated in cartilage growth or joint cavity formation were selected for expression analysis, comparing immobilised and control embryos. The candidate genes (Table 1) were also selected on the basis of some evidence of mechanosensitivity in another context (e.g. cell culture). Gene expression analysis was carried out on the same embryos used for morphometric analysis above; using the right hind limb for alcian blue staining/OPT scanning and the left hind limb for gene expression analysis. Three candidate genes, β1 integrin, FGFR2 and WNT9a showed no difference in expression pattern between control and immobilised specimens (not shown). Figure 6 shows differences in the expression observed in control and immobilised embryos on longitudinal sections through the knee joint (plane of section as in Figure 2D).

In the knee joint at this stage PTHLH expression was detected only at the very proximal and distal ends of the cartilaginous anlagen, restricted to the periarticular cartilage where the rudiments oppose (Figure 6A, A’). Immobilisation disrupts the characteristic pattern of PTHLH so that it is no longer restricted to the periarticular regions but is detected across the interzone (Figure 6B, B’) (n = 5/5). The expression pattern in immobilised animals appears to have “fuzzy” boundaries compared to the more clearly defined territories in control specimens. This surprising finding of expression across the joint region of a gene normally restricted to cells within cartilage rudiments is complimented by detection of COL2A1 transcripts in some cells spanning the interzone in immobilised individuals (n = 7/7), similar to the expression of PTHLP (Figure 6C, D, C’ D’).

Expression of FGF2 in control knee joints was detected within the chondrogenous layers of the interzone, part of the forming meniscus, the region of the future patella, prominently within the cranial cnemial crest (Figure 6E) and surrounding the developing tendons (not shown). Immobilisation resulted in a specific alteration to the spatial expression of FGF2 with expression no longer detected in the chondrogenous layers at the point where the femur is closest to the tibiotarsus (n = 5/5) (Figure 6F) i.e. normally continuous expression in the chondrogenous layers is disrupted. Expression in the presumptive patella region and meniscus was also absent in immobilised animals whereas expression in the cranial cnemial crest was unchanged (Figure 6F).

In control specimens BMP2 transcripts were detected in the perichondrium along the length of the rudiments, the intermediate layer of the interzone, the developing patella and joint capsule (Figure 6G). When BMP2 expression was analysed in immobilised embryos, clear, elevated expression was no longer detected in the intermediate layer (n = 10/10) (Figure 6H). Expression in the perichondrium and joint capsule remained unchanged and some expression of BMP2 within the patella could be detected although the expression level and size of the expression domain appeared to be reduced (not shown).

Similar to BMP2 expression, CD44 and HAS2 have very defined expression within the presumptive joint line region in the intermediate layer of the interzone (Figure 6I, k). HAS2 is also expressed in the most distal part of the inter-patella-femoral fat pad adjacent to the tibiotarsus (Figure 6I). CD44 also shows additional expression in the region of the future patella (Figure 6K), the muscle blocks and cells surrounding the ligaments (not shown). Expression of both genes was lost from the intermediate layer of immobilised joints (Figure 6J,L). This loss occurred in all specimens analysed (8 assayed for HAS2, 7 for CD44). Specific CD44 expression in the region of the future patella was lost in immobilised specimens (Figure 6L). In addition, both genes showed elevated expression throughout the inter-patella-femoral fat pad of immobilised embryos (Figure 6J,L).

## Discussion

Blocking muscle contractions in the chick embryo alters the biophysical environment of the developing musculoskeletal system. In this work we used computational modelling to demonstrate how rigid immobilisation would affect the mechanical stimuli generated in the developing knee joint and we investigated the impact of such immobilisation on the tissues of the developing joint at morphological and molecular levels. We showed that when dynamic stimulation is removed, patterning of the interzone and joint morphogenesis are altered with very specific changes to the shape of the cartilage rudiments and abnormal definition of tissue territories within the presumptive joint. The altered shape of the cartilaginous rudiments is accompanied by region specific changes in cell proliferation. The altered definition of tissues within the joint interzone is shown not only by the expression of tissue marker genes but also by altered expression of regulatory genes that are involved in steering differentiation and morphogenesis.

The dependence of correct joint development on stimulation from muscle contractions was previously shown by similar immobilisation studies in the chick [10], [11], [21], [23], [58] and using genetically altered mice that have absent, reduced or non-contractile muscle [19], [20]. However in the altered mechanical environment of mouse models, Nowlan et al [19] showed that forelimbs are more affected than hindlimbs and that while the elbow joint is severely affected, the knee joint appears normal. In the current work we compared the effects of immobilisation on the knee and elbow joints in the chick and found that the elbow joint showed similar but no more severe alterations (not shown) highlighting a clear and intriguing difference between avian and mammalian models. Computational modelling of the mouse model demonstrated that passive displacement of embryonic limbs *in utero* due to movement of the mother and normal littermates would produce greater stimulation of the hindlimbs than the forelimbs providing a possible source of compensation for the reduced stimulation, particularly in the hindlimbs (unpublished data). The *in ovo* situation of the chick embryo means less passive movement from external sources and therefore a greater reliance on muscle contractions to generate mechanical stimuli in the hindlimbs.

Previous studies demonstrated that knee joints in immobilised chick embryos fail to cavitate [10], [11], [21], [23], [58], [59], [60] but there has been very little emphasis on changes to the shape of the joint. Here we used morphometric analysis of 3D digital representations of the specimens to pin point the shape features of the distal femur that are dependent on extrinsically produced stimuli from muscle contractions, showing a link between the shape changes, changes in local cell proliferation and predicted biophysical stimuli. Specific changes included simplification of the shape of the medial and lateral condyles of the distal femur with flattening of the condyles and the absence of characteristic spurs. The strongest effect was seen on the separation of the condyles with a consistent narrowing of the intercondylar fossa. Cell division contributes to morphogenesis of a tissue when proliferation rates differ in a location specific manner and we previously showed that cell proliferation is greater in regions of the distal femur that grow most between stages HH30 and HH34 [30], for example the medial compared to the lateral condyle. Here we show that in immobilised embryos cell proliferation rates in the cartilaginous rudiments are reduced, specifically where dynamic mechanical stimulation is predicted to be strongest (dorsal aspect of both condyles and the region of intercondylar fossa) and where greatest shape changes are observed in immobilised specimens (reduction of height of the condyles and width of the intercondylar fossa). We therefore suggest that local patterns of biophysical stimuli contribute to the regulatory mechanisms controlling local growth. Previous *in vitro* studies have shown that mechanical stimulation can influence cell division and matrix biosynthesis [49], [50], [51], [53] but we show a location specific effect *in vivo*, relevant to morphogenetic changes. The altered mechanical environment of rigid paralysis might resemble that of a statically loaded culture. Static loads have been found to inhibit biosynthesis of articular cartilage while dynamic loading, such as in normal muscle contraction cycles, increases synthesis and proliferation of chondrocytes [50], [53]. Comparing the effects of rigid and flaccid paralysis (i.e. static compared to zero load), Osborne et al. [12] found that rigid paralysis led to a greater reduction in the width of the epipheses indicating that static loading may have an inhibitory effect on growth.

Currently much of what is known about cartilage growth in long bones relates to longitudinal growth and the associated regulation of the ossification process; very little is known about the control of local outgrowths and protrusions such as features of the condyles. Changes seen in the expression of PTHLP, FGF2 and BMP2 in immobilised animals have possible implications for the observed altered shape of the femoral condyles due to their roles in the regulation of diaphyseal cartilage growth [31]. PTHLP is known to maintain a pool of proliferating chondrocytes in the rudiments with the rate of chondrocyte proliferation within this pool modulated by BMPs and FGFs as part of a IHH/PTHLP feedback loop. Regional expression of these potential growth modulating molecules could influence local growth patterns within the femoral condyles. The altered expression of the genes encoding these molecules and our previous demonstration of mechanosensitivity of IHH expression in the developing tibiotarsus [29] indicate that mechanisms regulating cartilage growth are affected by immobilisation.

**Figure 7:**
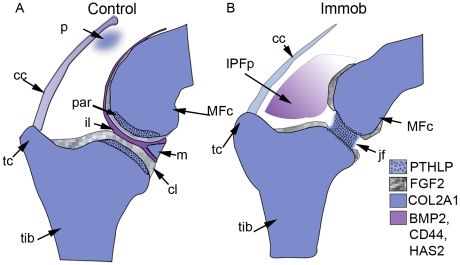
Representation of the altered patterns of regulatory gene expression due to immobilisation. Colour coded expression patterns of markers and regulatory genes of interest in both control and immobilised sections. cc; capsular condensation, cl; chondrogenous layer, il; intermediate layer, IPFp; inter-patella-femoral fat pad, jf; loss of joint line definition, m, meniscus, MFc; medial femoral condyle, p; patella, tc ; tibial crest, tib; tibiotarsus. Note: BMP2 and FGF 2 are expressed in the capsule (not shown in legend).

Immobilisation also impacted the process of cell differentiation in the interzone, as indicated by changes in characteristic gene expression patterns and histology. In the absence of normal muscle forces the expression patterns of marker genes Tenascin C and Collagen type II alpha 1 (COL2A1) and regulatory genes PTHLP, BMP2, FGF2, CD44 and HAS2 were altered (summarised in Figure 7). Normal expression of FGF2, BMP2, CD44 and HAS2 was disrupted or lost specifically in the interzone regions of immobilised embryos which acquire cartilage like tissue characteristics as indicated by the inappropriate activation of COL2A1 and PTHLP expression. In addition boundaries of gene expression between cartilage rudiments and the interzone were less distinct suggesting either cell movement and cell mixing between the territories or transdifferentiation of cells in the interzone to a cartilaginous character; the latter interpretation is supported by the findings of Kahn et al [20] of aberrant expression of COL2A1 in lineage labeled interzone cells (descended from Gdf5 expressing cells) in immobile mouse embryo limbs. Finite element models predicted that the patterns of biophysical stimuli created by muscle contractions correspond with the emergence of specific tissues in the joint, suggesting that they could contribute to the patterning of these tissues [30]. For example the chondrogenous layers which ultimately form the articular cartilages are predicted to develop under dynamic elevation of fluid velocity and stress. We propose from these findings that correct differentiation of interzone cells and the maintenance of interzone cell type are dependent on the mechanical environment to which they are exposed. It has been shown that cultured interzone cells initially express the interzone marker Gdf5, however after several days in culture the cells resemble chondrocytes and express markers such as Collagen type II [7]. This strongly supports the conclusion that following initial specification of the interzone, maintenance of the territory and further differentiation toward cell types of particular articular structures is dependent on mechanical stimulation.

Classical descriptions of joint development divide the process into two separate phases: interzone specification and cavitation. Earlier immobilisation studies suggested that only the cavitation phase is sensitive to mechanical stimulation since the interzone forms but fails to cavitate when contractions are altered [reviewed in 61,62]. Our findings show that under rigid paralysis the organisation of joint territories is altered as discussed above. Therefore mechanical stimulation impacts cellular processes involved in the definition of tissues and cellular differentiation prior to cavitation. This shows that mechanical stimulation should not be seen as having a molding effect on intrinsic morphogenetic processes [62], [63] but as influencing the processes fundamentally. It also shows the importance of viewing joint development as a series of interlinked events, as argued by Lambe et al [61]; events that are impacted by mechanical stimulation from an early stage.

The data presented here show that multiple aspects of knee joint patterning and morphogenesis are affected when mechanical stimulation is altered (cell proliferation, cell differentiation and tissue boundaries with consequential alterations to the shape of rudiment epiphyses and the structure of the joint and associated tissues). These compound effects support our previously proposed hypothesis [30] that local patterns of biophysical stimuli create a type of positional information that contributes to the correct patterning of emerging tissues in the joint. Finite Element analysis was previously used to predict patterns of biophysical stimuli in the normal developing joint and here we used the same approach to simulate rigid paralysis showing changes in the stimuli patterns that correspond with the major changes observed in immobilised embryos. In rigid paralysis all muscles are in tetanus. Modelling this situation showed a reduction of stimuli in femoral condyles corresponding to sites of reduced proliferation and shape change and the replacement of a complex dynamic pattern of stimuli by a simplified, static environment. The observed changes in growth and proliferation may arise because of either the reduced stimulation or the lack of dynamic stimulation or a combination of both. In the interzone the patterns of stimuli predicted under normal and immobilised situations is similar but of course the forces are static rather than dynamic underlining the importance of dynamic stimuli, also observed in the response of cells in culture [49], [50]. However, alterations to the stimuli patterns may be greater than predicted here for a number of reasons. The rigid model was based on normal morphology and assumed normally functioning tendons but tendon development is known to be negatively affected by immobilization [64] so transfer of loads may be compromised. Also the altered shape of the rudiments in immobilized specimens may alter the forces but this is unlikely to have a large effect since we previously found that the general pattern of the forces do not change dramatically with shape changes over time [30]. So our predictions may be conservative and actual changes to the biophysical environment in immobilized embryos may be more extreme than predicted.

Despite numerous examples of mechanoresponsiveness of tissues and cells and a long list of mechanosensitive genes demonstrated in culture, with a growing number demonstrated *in vivo* [reviewed in 65], we know very little about the biological mechanisms that integrate biophysical stimuli with gene regulation. Several possible sensory mechanisms including integrins, stretch activated ion channels and the primary cilium have been indicated in cellular mechanotransduction [66], [67], [68], [69], [70]. A number of proteins have been shown to be phosphorylated as a result of mechanical stimulation including MAP kinases like ERK1/2 [71]. Also, mechanical stimulation of human adult articular chondrocytes in culture results in transient tyrosine phosphorylation of the protein kinase pp125FAK, the focal adhesion protein paxillin, and the multifunctional signaling molecule b-catenin [67]. Primary Cilia have also been proposed as a cellular antenna capable of detecting mechanical strains [72], [73] and have been identified on both adult and embryonic chondrocytes [74], [75], [76]. A number of ECM receptors are expressed on cilia [77] leading to the proposal that cilia may transduce mechanical forces from the ECM to the cell. Primary cilia are particularly interesting in the present context because of the association between cilia and hedgehog signaling [78].

The regulatory genes analysed in this study were previously defined as mechanosensitive based on *in vitro* assays (Table 1). Here we show that spatial restriction of the gene expression patterns of PTHLP, BMP2, FGF2, HAS2, CD44, COL2A1 and TNC is responsive to biophysical stimuli in an *in vivo* context (Figure 7) revealing potential key mediators of mechanical stimulation of joint development that warrant closer analysis. A key question is if the genes respond directly to mechanical stimuli or if they lie downstream of other more direct mediators. If we can demonstrate in an appropriate assay system that the response is direct we open the possibility of revealing the cellular mechanisms that links mechanical stimulation with gene regulation. This is a key focus of future work.

Understanding the input of mechanical signals in the stable differentiation of skeletal tissues is of particular importance in attempts to regenerate tissue for replacement therapies including therapies for patients with articular cartilage defects such as arthritis. A wide range of different stimuli and culture methods have been used to recapitulate the process of articular cartilage development with varying success [69], [79], [80]. One particular problem is preventing chondrogenic cells from undergoing hypertrophy as they would in endochondral ossification [81]. A better understanding of how the interzone develops and in particular the mechanical requirements for articular cartilage development in embryos provides useful information on the type and magnitude of loads which could be applied to cultures to produce cartilage of the appropriate type and quality for regenerative therapies. The present work indicates that conditions that increase interstitial fluid flow might be beneficial in the regeneration of articular cartilage. To recapitulate stable cartilage differentiation, the process needs to be better understood.

## Materials and Methods

### *In ovo* immobilisation

Fertilised chick eggs were purchased from Enfield Broiler Breeders and incubated (Solway Natureform) at 37.5°C and 70% humidity. The work was approved by the ethics committee Trinity College Dublin. Work on early chick embryos in ovo does not require a license from the Irish Ministry of Health under European Legislation. Immobilisation was induced by the application of the neuromuscular blocking agent Decamethonium bromide (DMB) (Sigma). Following 3 days of incubation, 4 mls of albumen was removed from each egg using a 21 gauge needle. Immobilisation treatments consisted of the application of 100 µl of 0.5% DMB (Sigma) in sterile Hank's Buffered Saline Solution (HBSS) (Sigma) plus 100 units/ml antibiotic/antimycotic (Gibco) were started after 4.5 days of treatment. Controls were treated with 100 µl of sterile HBSS. Treatment was repeated daily until the embryos were harvested after a further 4 or 5 days of incubation. The experiment was repeated independently three times.

At the end of the experiment the embryos were dissected in ice cold Phosphate Buffered saline (PBS) (Sigma), staged using the Hamburger and Hamilton criteria [56] and cut longitudinally down the spine. The right side was fixed overnight in fresh 4% paraformaldehyde (PFA) in PBS at 4°C, dehydrated through a graded series of methanol/PBT (0.1% Triton X100 in PBS; 25%, 50%, 75% methanol; 1×10 minute) washes, followed by 2×10 minutes 100% methanol. After dehydration the embryos were stored in 100% methanol at −20°C until needed. The left hand side of the embryo including the head and neck was fixed in 95% ETOH for 3 days and stained for cartilage using alcian blue [as per 5]. The knee joint region were subsequently imaged using Optical Projection Tomography [as per 5].

### *In situ* hybridisation

Expression probes were prepared from cDNA clones obtained from the Biotechnology and Biological Sciences Research Council (BBSRC) ChickEST Database and its bank of expressed sequence tags (ESTs) [82]. Details of the clones used to produce all probes are given in Table 3. Antisense and sense digoxigenin-labelled RNA was transcribed *in vitro* from 1 µg of linearized plasmid using T7 and T3 promoter sites (according to insert orientation) in the pBluescript II KS+ vector with all components for *in vitro* transcription purchased from Roche, Germany. DNA template was degraded by incubation of probes with RNase free DNase (Roche) and probes were purified on G25 columns (Amersham Biosciences, USA) according to the manufacturer's instructions. Probe concentrations were determined by spectophotometry and probes were stored at 20°C.

**Table 3:** Summary of in situ probes and elected ChickESTs.

Gene	ChEST reference	Genebank reference	Alignment of ChEST on reference
BMP2	ChEST 367 j4	AY237249.1	66–709
CD44	ChEST 343 m10	XM_001232450.1	1940–1794
HAS2	ChEST 500 e4	NM_204806.1	2507–2025
β1 integrin	ChEST 500 j17	NM_001039254.1	270–1022
WNT9a	ChEST 592 n13*	NM_204891.1	784–1213
PTHLP	ChEST 533 c1	AB175678	68–734
FGF2	ChEST 432 i3	M95706.1	145–445
FGFR2	ChEST 699 l24*	NM_205319.1	1969–2716
COL2A1	ChEST 179 l15	NM204426.1	3887–4689
TNC	ChEST 681 l9	CHKTEN	2809–2075

Limbs fixed in 4%PFA, dehydrated and stored at −20°C were rehydrated through a series of methanol/PBT solutions (75%, 50%, 25%; each 10 minutes) at 4°C and subsequently washed 2×10 minutes in PBT. On rehydration the limbs were further dissected to remove the foot and skin. The knee joint of the specimens were was then embedded in 4% low melting point (LMP) agarose/PBS (Invitrogen, UK) and 100 µm longitudinal sections were cut using a vibrating microtome (VT1000S, Leica). Hybridisation was carried out largely as per Nowlan et al [13].

### Histology

Hind limbs of immobilised and control embryos, 4%PFA fixed, dehydrated and stored at −20°C, were embedded in paraffin wax and sectioned as described in Roddy et al 2009, stained using 0.5% Alcian blue (30 min), Harris Haematoxylin (6 min) (Sigma-Aldrich) and counterstained using Eosin. The sections were mounted and photographed using a Nikon Optiphot-2 microscope mounted with a Canon EOS 350D camera.

### Determining rates of cell proliferation

The proportion of proliferating cells was determined using the mitosis marker anti-phospho-histone H3 PABs (Millipore). Briefly, the hind limbs of control and immobilised embryos (n = 3) were rehydrated, skinned and embedded in 1.5% agarose, 5% sucrose. The blocks were equilibrated in 30% sucrose solution and frozen over a dry ice bath. 25–30 µm longitudinal sections were collected on BDH superfrost+ slides.

Following heat mediated antigen retrieval (50 µM Tris pH 8 for 35 minutes in a 95°C water bath), sections were washed (3X PBS with 0.5% Triton X-100 and 0.1% Tween 20) and blocked in 5% normal goat serum in 0.5% Triton X-100 and 0.1% Tween 20 for one hour at room temperature. Incubation with the primary antibody (anti-phospho-histone H3 PABs, Millipore P84243), was carried out in blocking solution overnight at 4°. Sections were washed and blocked as before and incubated in secondary antibody Cy3 goat anti-rabbit IGg (1/200, Jackson immuno) at 4°C overnight. Following further washing, the sections were mounted in ProLong Gold anti-fade reagent with DAPI (4′, 6-diamidino 2-phenylindole) (Invitrogen).

The density of proliferating chondrocytes was determined in five cartilage regions (Figure 3g): adjacent to the intercondylar fossa, and the dorsal and ventral portions of the medial and lateral condyles. Each region was imaged separately using an Olympus FV1000 point scanning confocal microscope. The numbers of chondrocytes and proliferating cells were counted within a box 1.44 mm^2^ for two independent focal planes on two sections per specimen (n = 3). The effect of treatment on the cell proliferation was statistically analysed using a generalised linear mixed effects model where multiple sections were nested within individuals in order to take account of the nested nature of the data (R statistical package).

### Modelling rigid limb paralysis using FE analysis

DMB induces rigid paralysis where the muscles are in continuous contraction [12]. To account for this, the previously described Finite Element models of the developing knee [30] were adjusted to simulate rigid muscle paralysis by applying all muscle forces simultaneously and continuously. In the normal model which represents contractions in the control experimental situation, the muscles attached to the ventral tibiotarsus and fibula were active during the flexion contraction while those attached to the capsular condensation are activate in the extension contraction. Immobilisation leads to a reduction in muscle size and its ability to transmit forces [83], [84]. The magnitudes of the forces applied to the model were therefore adjusted to 75% of the normal estimation [derived from 84]. Reiser et al. [84] measured the forces generated by normal and immobilised embryonic muscles. The same boundary conditions and material properties were used in normal and paralysis models.
